# Supplementary material for: IGF1R regulates retrograde axonal transport of signalling endosomes in motor neurons
Source: EMBO Rep. 2020 Feb 7;21(3):e49129. doi: 10.15252/embr.201949129 (PMC7054680; doi:10.15252/embr.201949129)
Supplement: Supplementary file 1 — Appendix [file EMBR-21-e49129-s001.pdf]

Appendix- Fellows et al, 2020

TABLE OF CONTENTS

*Appendix Figure S1- The IGF1R pathway influences retrograde axonal transport of signalling endosomes* ........ 2

*Appendix Figure S2- Akt inhibition increases the velocity of retrograde signalling endosomes* ........ 3

*Appendix Figure S3- IGF1R modulation has no effect on microtubule post-translational modifications* ........ 5

*Appendix Figure S4- IGF1R activity does not affect axonal transport of lysosomes* ........ 7

*Appendix Figure S5- IGF1R modulation has no effect on the expression of cytoplasmic dynein or Kinesin-1* ........ 9

*Appendix Figure S6- Example images from puromycin-PLA* ........ 10

*Appendix Figure S7- IGF1R inhibition increases the rate of retrograde axonal transport in an ALS mouse model* ........ 11

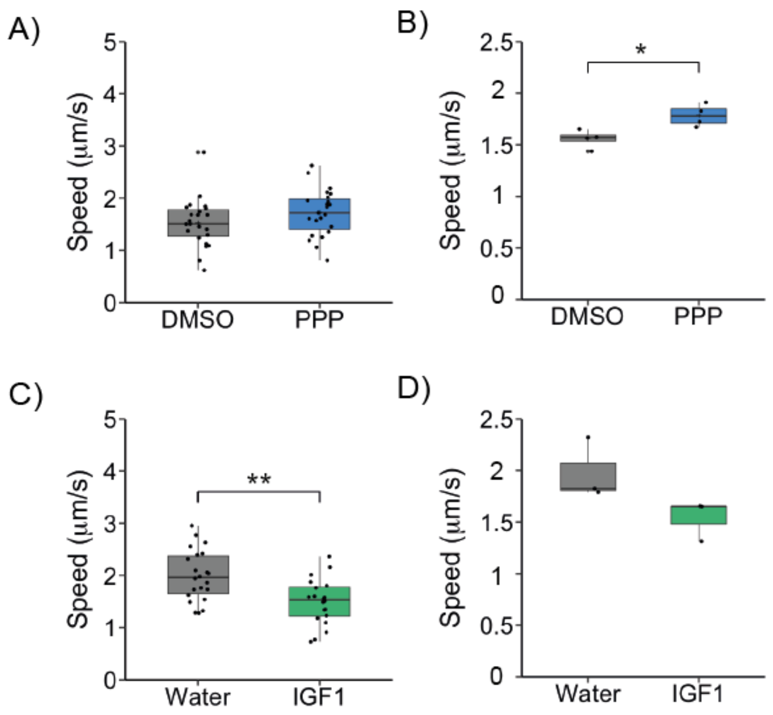


APPENDIX FIGURE S1- THE IGF1R PATHWAY INFLUENCES RETROGRADE AXONAL TRANSPORT OF SIGNALLING ENDOSOMES

A) Graph shows the average velocity of $H_cT$-containing organelles per axon after PPP treatment (P=0.15, Student's T-test, N=4 independent experiments, boxplot shows median, first and third quartiles. Upper/lower whiskers extend to 1.5 * the interquartile range, boxplot shows median, first and third quartiles).

B) Graph shows the average velocity of $H_cT$-containing organelles per experiment after PPP treatment (* P=0.017, Student's T-test, N=4 independent experiments, boxplot shows median, first and third quartiles. Upper/lower whiskers extend to 1.5 * the interquartile range).

C) Graph shows the average velocity of $H_cT$-containing organelles per axon after IGF1 treatment (** P=0.001, Student's T-test, N=3 independent experiments, boxplot shows median, first and third quartiles. Upper/lower whiskers extend to 1.5 * the interquartile range).

D) Graph shows the average velocity of $H_cT$-containing organelles per experiment after IGF1 treatment (P=0.09, Student's T-test, N=3 independent experiments, boxplot shows median, first and third quartiles. Upper/lower whiskers extend to 1.5 * the interquartile range).

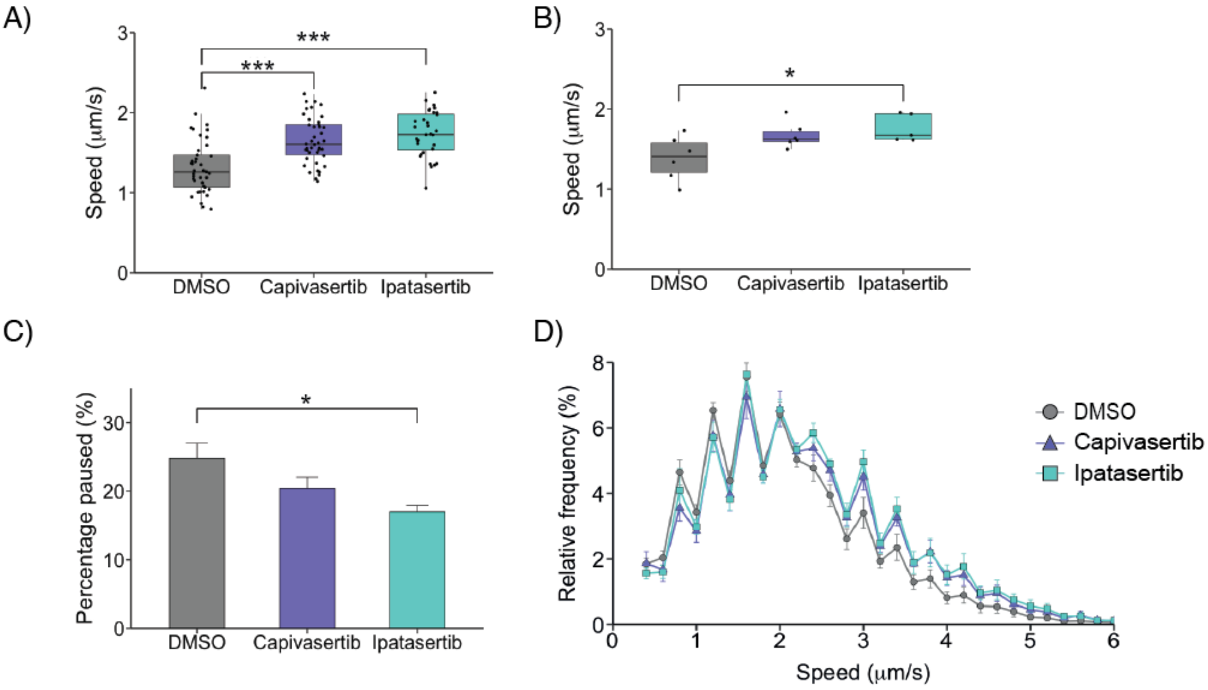


APPENDIX FIGURE S2- AKT INHIBITION INCREASES THE VELOCITY OF RETROGRADE SIGNALLING ENDOSOMES

A) Graph shows the average velocity of $H_cT$-containing organelles per axon after Capivasertib and Ipatasertib treatment for 45 mins before imaging (DMSO: 483 endosomes, 34 axons, 41,897 movements; Capivasertib: 658 endosomes, 43 axons, 49,841 movements; Ipatasertib: 605 endosomes, 32 axons, 41,485 movements, *** P=$2.44x10^{-7}$, One-way ANOVA, Tukey's post-hoc test: DMSO-Capivasertib *** P=$8.9x10^{-5}$, DMSO-Ipatasertib *** P=$2.5x10^{-5}$, N=6 independent experiments, boxplot shows median, first and third quartiles. Upper/lower whiskers extend to 1.5 * the interquartile range).

B) Graph shows the average velocity of $H_cT$-containing organelles per experiment after treatment with Capivasertib and Ipatasertib for 45 mins before imaging (* P=0.025, One-way ANOVA, Tukey's post-hoc test: DMSO-Capivasertib P=0.081, DMSO-Ipatasertib * P=0.028; N=6 independent experiments, boxplot shows median, first and third quartiles. Upper/lower whiskers extend to 1.5 * the interquartile range)

C) Graph shows the average pausing of $H_cT$-containing organelles per experiment after Capivasertib and Ipatasertib treatment for 45 mins before imaging (* P=0.027, One-way ANOVA, Tukey's post-hoc test: DMSO-Capivasertib P=0.20, DMSO-Ipatasertib * P=0.022, N=6 independent experiments, data shown are mean ± SEM).

D) Speed distribution profile of Capivasertib- and Ipatasertib-treated neurons and controls. Both compounds caused an increase in instantaneous velocities of $H_cT$-containing organelles compared to controls (N=6 independent experiments , data shown are mean ± SEM).

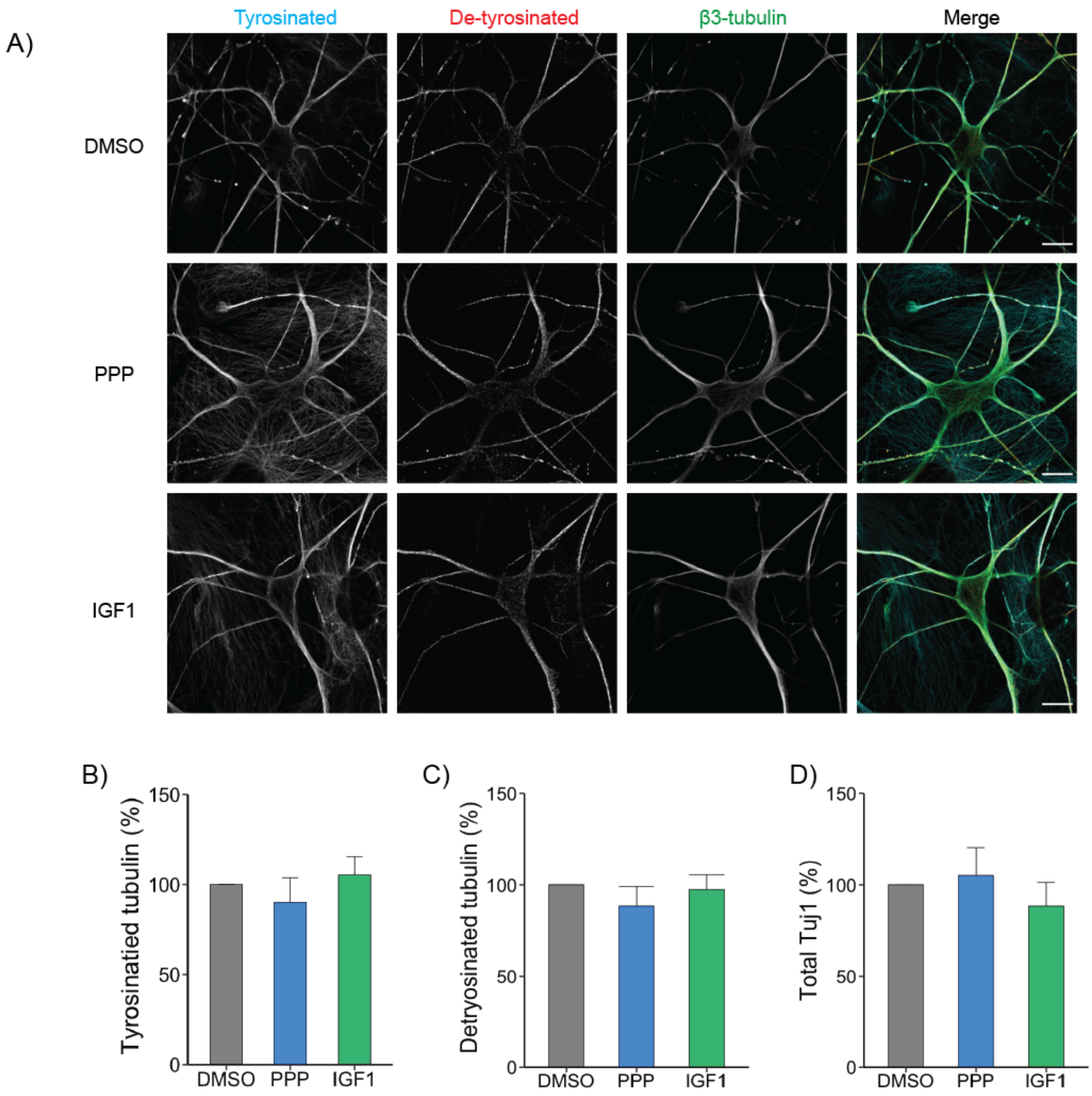


APPENDIX FIGURE S3- IGF1R MODULATION HAS NO EFFECT ON MICROTUBULE POST-TRANSLATIONAL MODIFICATIONS

A) Staining of tyrosinated and detyrosinated $\alpha$-tubulin and $\beta$-tubulin in PMN after treatment with 1 $\mu$M PPP or 50 ng/ml IGF1 for 60 min. The scale bar is 10 $\mu$m.

B) Quantification of tyrosinated $\alpha$-tubulin levels from A). Results were normalised relative to $\beta$-tubulin. IGF1R modulation had no effect on the amount of tyrosinated $\alpha$-tubulin (DMSO- 100%, IGF1- 105.3 $\pm$ 9.9%, PPP- 89.9 $\pm$ 13.8%, P=0.55, one-way ANOVA, N= 4 independent experiments, 31-32 cells per condition, data shown are mean $\pm$ SEM).

C) Quantification of detyrosinated α-tubulin levels from A). Results were normalised relative to β-tubulin. There was no change after treatment (DMSO- 100%, PPP-88.2 ± 10.9%, IGF1- 97.5 ± 8.0%, P=0.55, one-way ANOVA, N=4 independent experiments, 31-32 cells per condition, data shown are mean ± SEM).

D) Quantification of β-tubulin levels from A). Treatment with PPP or IGF1 did not affect the amount of β-tubulin present in PMN (DMSO-100%, PPP- 105 ± 15.2%, IGF1- 87.9 ± 13.1%, P=0.59, one-way ANOVA, N=4 independent experiments, 31-32 cells per condition). Data shown are mean ± SEM.

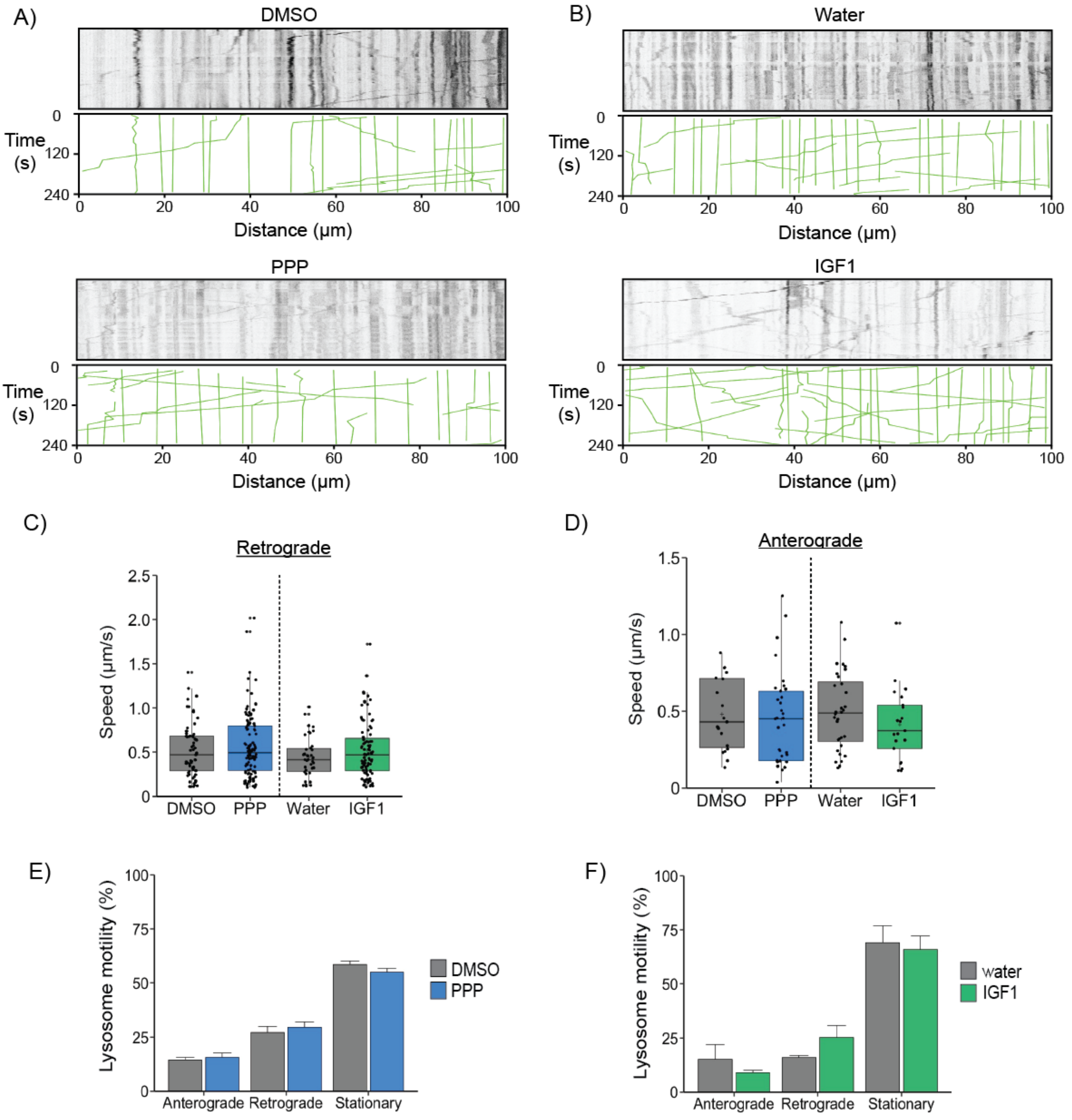


APPENDIX FIGURE S4- IGF1R ACTIVITY DOES NOT AFFECT AXONAL TRANSPORT OF LYSOSOMES

A) Kymographs of LysoTracker$^{TM}$ Green DND-26 labelled lysotracker in DIV 6 PMN after treatment with 1 $\mu$M PPP or control for 45 min.

B) Kymographs of LysoTracker$^{TM}$ Green DND-26 labelled lysotracker in DIV 6 PMN after treatment with 50 ng/ml IGF1 or control for 45 min.

C) Analysis of IGF1R modulation on retrograde lysosomal velocity. Neither PPP or IGF1 treatment had a significant effect on retrograde transport of lysosomes (DMSO: 0.53 ± 0.04 μm/s, cargo- 65, N=2 independent experiments; PPP: 0.57 ± 0.03 μm/s, cargo- 112, N=3 independent experiments; P= 0.41, Student's T-test; water: 0.44 ± 0.04 μm/s, cargo- 38, N=3 independent experiments; IGF1: 0.52 ± 0.03 μm/s, cargo- 91, N=3 independent experiments, P= 0.13, Student's T-test, boxplot shows median, first and third quartiles. Upper/lower whiskers extend to 1.5 * the interquartile range).

D) Analysis of IGF1R modulation on anterograde lysosomal velocity. PPP and IGF1 caused no significant change in anterograde transport rates (DMSO: 0.48 ± 0.05 μm/s, cargo- 19, videos- 13, N= 2 independent experiments; PPP: 0.4 ± 0.05 μm/s, cargo- 33, videos- 22, N= 3 independent experiments; P= 0.68, Student's T-test; water: 0.49 ± 0.04 μm/s, cargo- 32, N=3 independent experiments video- 13; IGF1: 0.41 ± 0.05 μm/s, cargo- 21, N=3 independent experiments, videos-21; P= 0.22, Student's T-test, boxplot shows median, first and third quartiles. Upper/lower whiskers extend to 1.5 * the interquartile range).

E) Analysis of the directionality of lysosome transport. There was no change in the directionality of lysosome transport after PPP treatment compared to controls (anterograde: DMSO- 14.4 ± 1.2%, cargo- 48; PPP- 15.6 ± 2.1%, cargo- 81, P= 0.65; retrograde: DMSO-27 ± 2.8%, cargo-88, PPP- 29.4 ± 2.5%, cargo- 160, P= 0.58; stationary: DMSO-58.6 ± 1.6%, cargo- 194; PPP- 55 ± 1.8%, cargo- 295, P= 0.24, Student's T-test, N=2 for DMSO and N=3 for all other conditions, data shown are mean ± SEM).

F) Analysis of the directionality of lysosome transport. IGF1 didn't change the directionality of lysosomes after treatment. Quantified from B) (anterograde: water- 15.1 ± 6.9%, cargo- 55; IGF- 8.9 ± 1.1%, cargo- 39, P= 0.53; retrograde: water- 15.9 ± 1.0%, cargo- 55, IGF-25.3 ± 5.4%, cargo- 120, P= 0.22; stationary: water- 69 ± 7.9%, cargo- 232, IGF- 65.8 ± 6.3 %, cargo-290, P= 0.783, Student's T-test, N=2 for DMSO and N=3 for all other conditions, data shown are mean ± SEM).

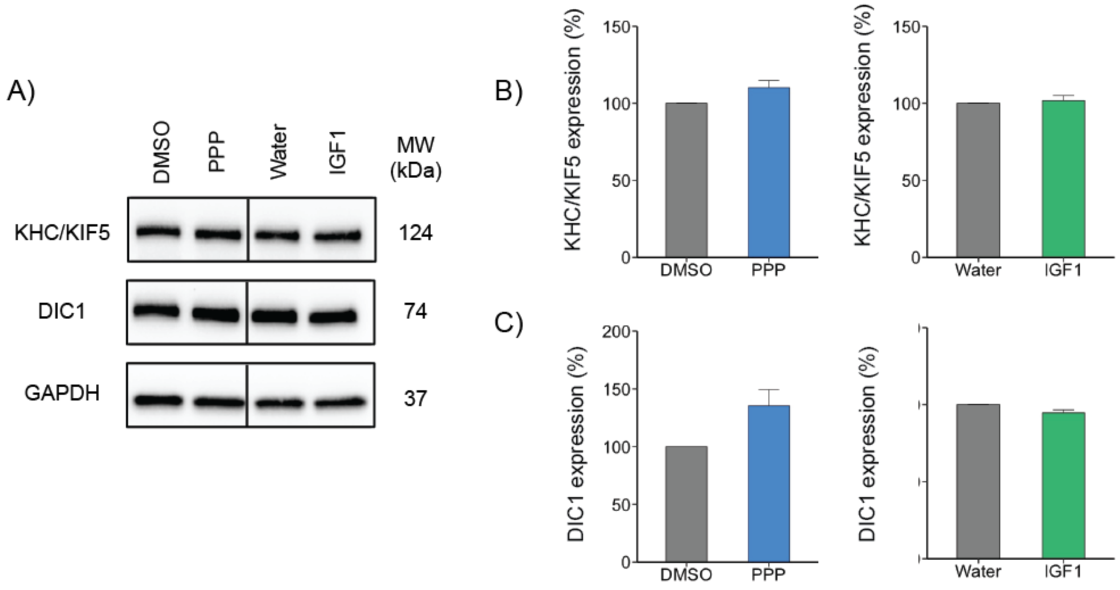


<u>APPENDIX FIGURE S5- IGF1R MODULATION HAS NO EFFECT ON THE EXPRESSION OF CYTOPLASMIC DYNEIN OR KINESIN-1</u>

A) PMN were treated with 1 μM PPP or 50 ng/ml IGF1 for 60 min and the expression of dynein intermediate chain (DIC1) and kinesin-1 (KHC/KIF5) were assessed via western blot. All proteins were assessed on the same blot along with proteins in Fig5.

B and C) Quantification of western blots in A). Protein levels were normalised to GAPDH. There was no significant change in levels of both motor proteins after treatment with PPP (KHC/KIF5: DMSO-100%, PPP-110.2 ± 4.7%, N=3 independent experiments, P=0.16; DIC1: DMSO-100%, PPP-135 ± 14.1%, N=5 independent experiments, P=0.067, Student's T-test) or IGF1 (KHC: water-100%, IGF1-101.76 ± 3.2%, N=3, P=0.63; DIC: water-100%, IGF1-94.7 ± 2.1%, N=3 independent experiments, P=0.12, Student's T-test, data shown are mean ± SEM).

A)

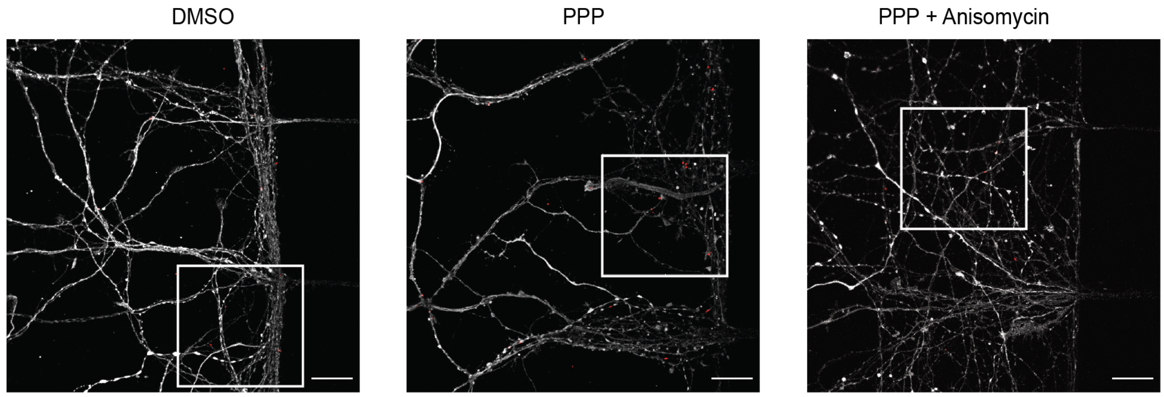


APPENDIX FIGURE S6- EXAMPLE IMAGES FROM PUROMYCIN-PLA

A) Puromycin-PLA images from the axonal compartment of PMN treated with PPP grown in microfluidic devices. White box indicates the zoomed region shown in Fig 6. Scale bars are 25 $\mu$m. White- $\beta$3-tubulin.

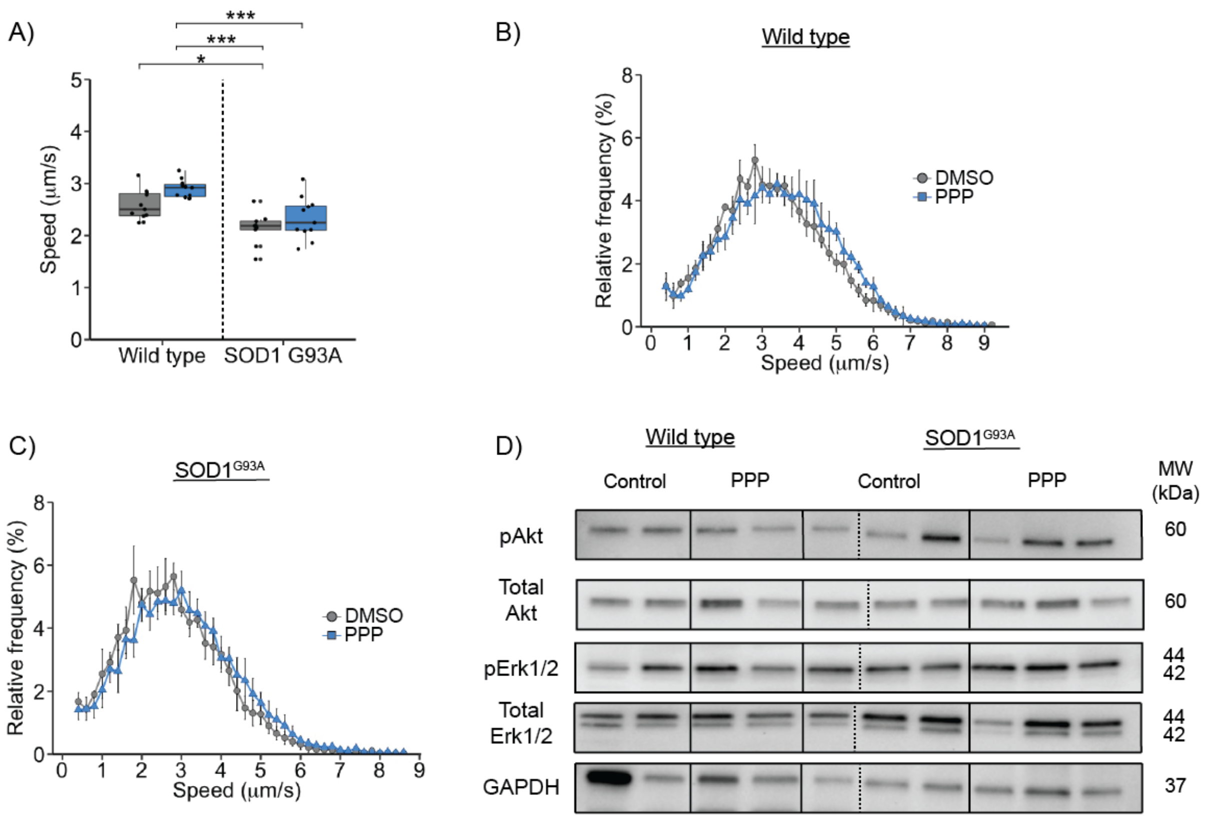


## Appendix Figure S7- IGF1R inhibition increases the rate of retrograde axonal transport in an ALS mouse model

A) The graph shows the average velocity of $H_CT$-containing organelles per video (P=0.019 (treatment), P= $3.83\times10^{-6}$ (genotype), two-way ANOVA, Tukey's post-hoc text, WT-control: SOD1$^{G93A}$- control, * P=0.01 ;WT-control: WT-PPP, P=0.09; WT-PPP: SOD1$^{G93A}$- control, *** P=0.00001; WT-PPP: SOD1$^{G93A}$-PPP, *** P=0.0005, N=4 independent experiments for all conditions).

B) and C) Speed distribution graphs of wild type and SOD1$^{G93A}$ mice treated with PPP (blue squares) or vehicle control (grey circles). Graphs show increase in instantaneous velocities in both wild type and SOD1$^{G93A}$ mice. N=4 independent experiments. Data shown are mean ± SEM.

D) Example western blot of sciatic nerve lysates from wild type and SOD1$^{G93A}$ mice treated with either PPP or vehicle control were probed for pAKT (Ser473), total Akt, pErk1/2 (Thr 202/ Tyr 204) and total Erk1/2. Dashed line indicated where blots have been joined.
