# Supplementary material for: IGF1R regulates retrograde axonal transport of signalling endosomes in motor neurons
Source: EMBO Rep. 2020 Feb 7;21(3):e49129. doi: 10.15252/embr.201949129 (PMC7054680; doi:10.15252/embr.201949129)
Supplement: Supplementary file 2 — Expanded View Figures PDF [file EMBR-21-e49129-s002.pdf]

Expanded View Figures

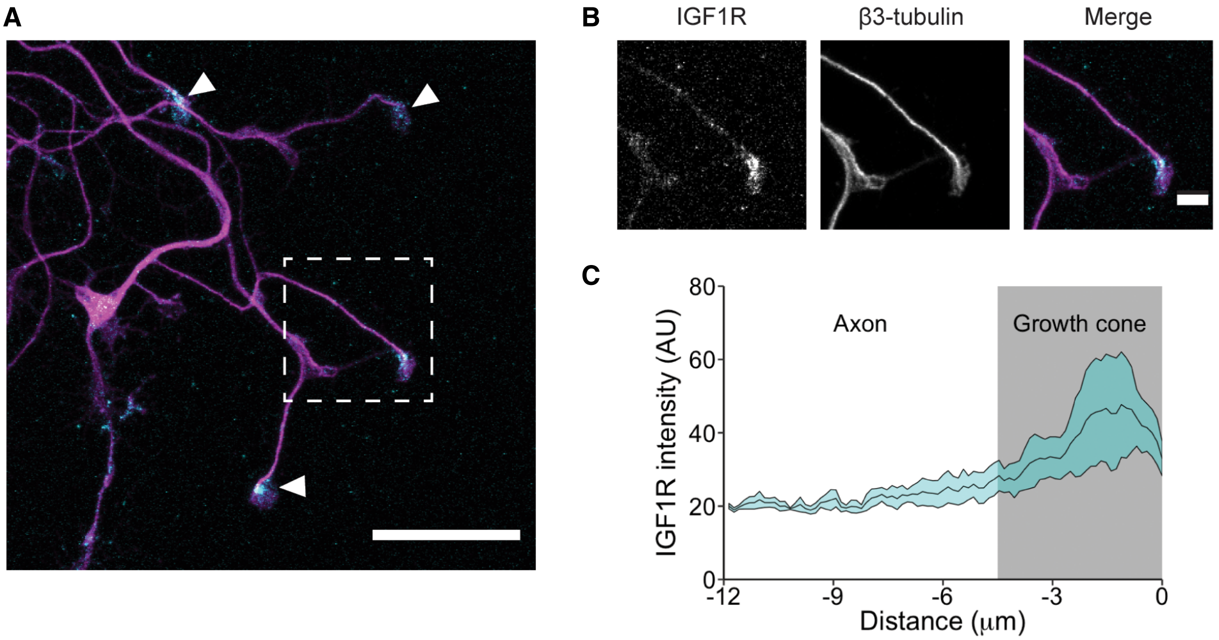

Figure EV1. IGF1R localizes to the axonal growth cone.

A Example image of DIV6 PMN labelled with β3-tubulin and IGF1R. White arrowheads point to axonal growth cones. Box shows zoomed region in B). The scale bar length is 20 µm.

B IGF1R staining at the growth cones of axons. The scale bar length is 4 µm.

C Quantification of IGF1R intensity along the axon. IGF1R accumulates at the growth cone of axonal processes (72 cells, *N*=3 independent experiment). All data shown are mean ± SEM.

Figure EV2. PPP downregulates Akt signalling.

A pAkt staining in PMN treated with PPP or IGF1. The scale bar is 10 µm.

B Image quantification. PPP treatment reduces the mean pAkt intensity in PMN (**P* = 0.046, Student's *t*-test, 32 cells for each condition, *N* = 3 independent experiment). IGF1 caused no significant change in pAkt levels in PMN (*P* = 0.25, Student's *t*-test, 32 cells for each condition, *N* = 3 independent experiments). All data shown are mean ± SEM.

C Representative Western blot showing the level of TSC2 phosphorylation in PMN and N2A cells after 1 µM Capivasertib and Ipatasertib treatment for 60 min. GAPDH was used as a loading control.

D Quantification of Western blot data. PMN and N2A cells were treated with either 1 nM, 10 nM, 100 nM or 1 µM of Capivasertib and Ipatasertib for 60 min. PMNs show decreased level of TSC2 phosphorylation after treatment. Capivasertib and Ipatasertib (1 µM) are *N* = 2 independent experiments. All other conditions are *N* = 3 independent experiments; data shown are mean ± SEM.

E Quantification of Western blot data. N2A shows decreased level of TSC2 phosphorylation after treatment with Capivasertib and Ipatasertib. Capivasertib 10 nM and 100 nM are *N* = 3 independent experiments; all other conditions are *N* = 4 independent experiments; data shown are mean ± SEM.

© 2020 The Authors

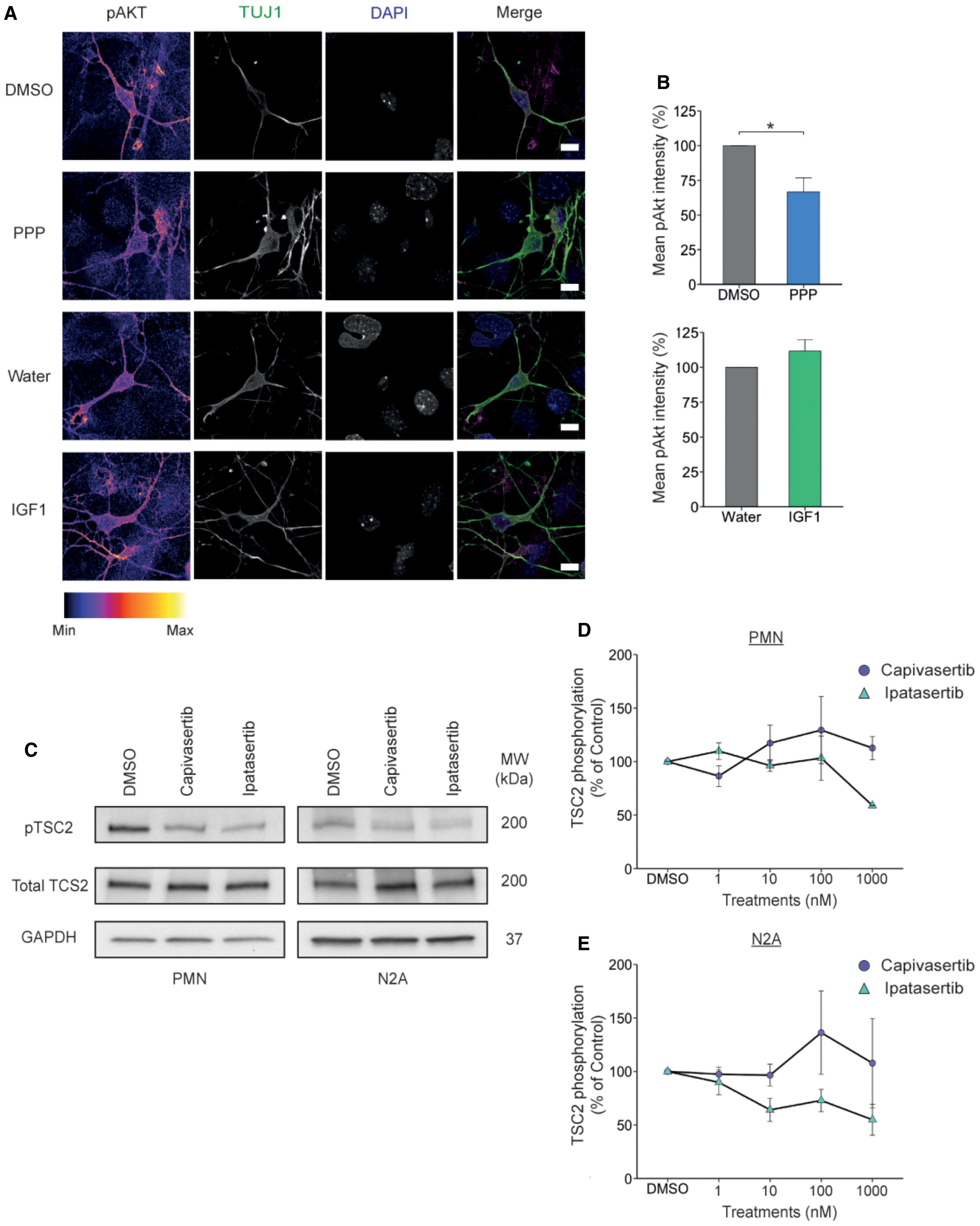

Figure EV2.

© 2020 The Authors

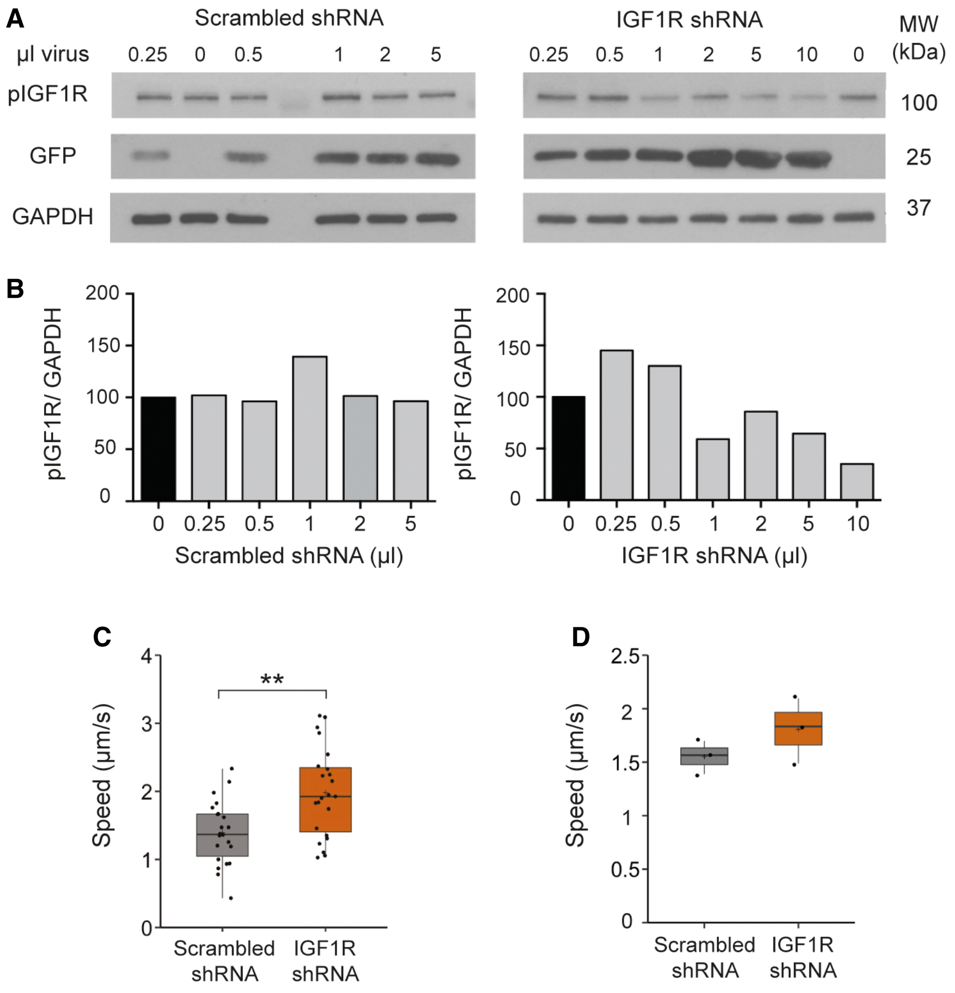

Figure EV3. shRNA knockdown of IGF1R in PMN.

A Western blot of pIGF1R after lentiviral delivery of a shRNA targeting IGF1R or scrambled control in PMN. Cells were lysed 6 days after treatment.

B The quantification of the Western blots in A). The shRNA viral construct effectively decreases levels of pIGF1R compared to scrambled control (*N* = 1 independent experiments).

C Graph shows the average velocity of H_CT-containing organelles per axon after IGF1R knockdown (***P* = 0.001, Student's *t*-test, *N* = 3 independent experiments; boxplot shows median, first and third quartiles. Upper/lower whiskers extend to 1.5 * the interquartile range).

D Graph shows the average velocity of H_CT-containing organelles per experiment after IGF1R knockdown (*P* = 0.26, Student's *t*-test, *N* = 3 independent experiments; boxplot shows median, first and third quartiles. Upper/lower whiskers extend to 1.5 * the interquartile range).

© 2020 The Authors

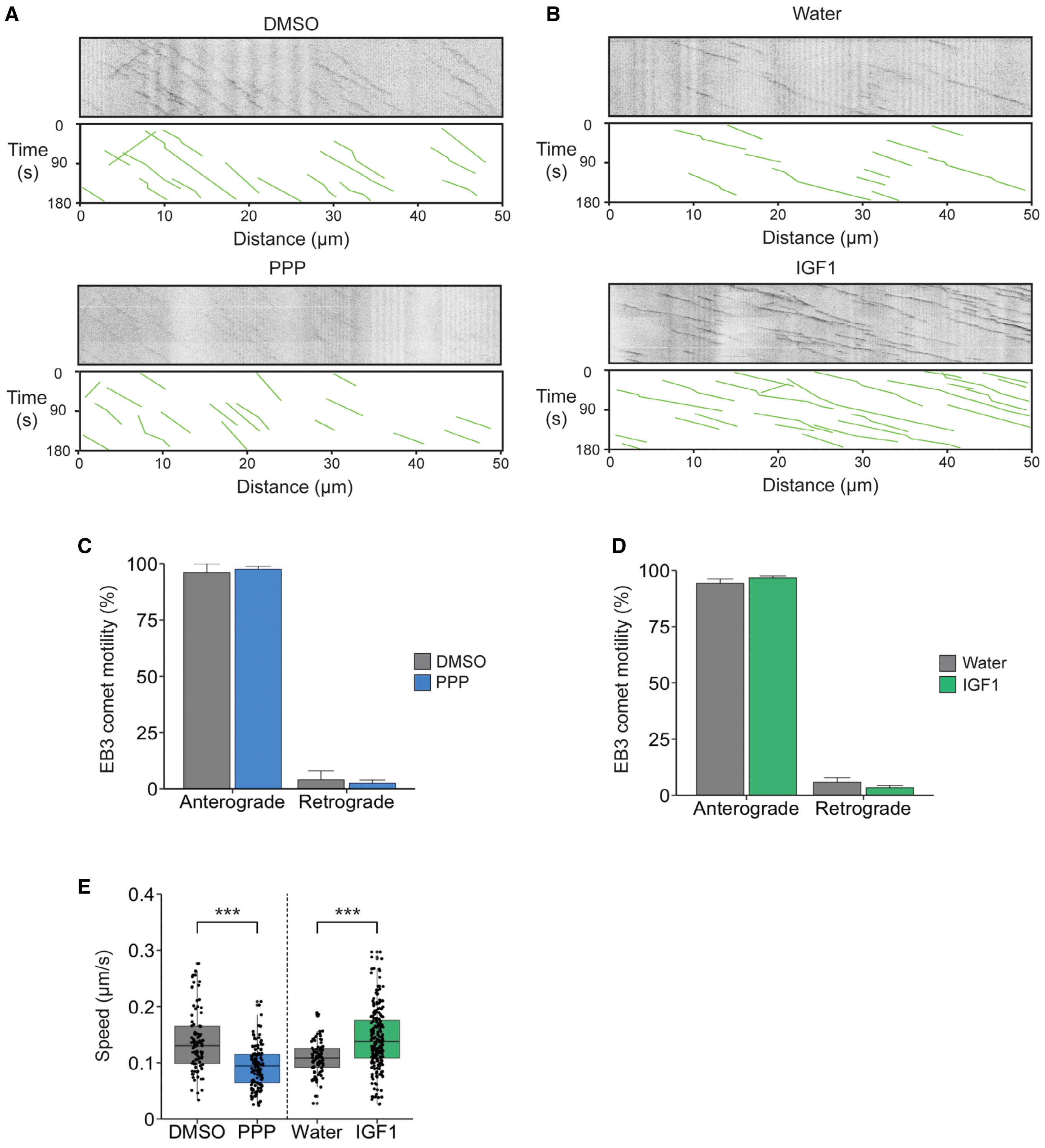

Figure EV4.

 © 2020 The Authors

◀ **Figure EV4. IGF1R influences microtubule dynamics but does not alter polarity.**

A Kymographs of GFP-EB3 comets after treatment with 1 μM PPP or DMSO for 45 min.
B Kymographs of GFP-EB3 comets after treatment with 50 ng/ml IGF1 or water for 45 min.
C Quantification of EB3-GFP directionality after PPP treatment. There was no change in microtubule polarity (anterograde: DMSO- 96.1 ± 3.9%; PPP- 97.6± 1.3%, $P = 0.77$; retrograde: DMSO-3.9 ± 3.9% versus 2.4 ± 1.3%, $P = 0.77$, Student's t-test, $N = 4$ independent experiments (PPP), $N = 2$ independent experiments (DMSO); data shown are mean ± SEM).
D Quantification of EB3-GFP directionality after IGF1 treatment. IGF1 also caused no change in polarity of new microtubules (anterograde: water- 96.6 ± 1.0%; IGF1- 94.2 ± 2.0%, $P = 0.23$; retrograde: water- 3.4 ± 1.0% versus 5.8 ± 2.0%, $P = 0.23$, Student's t-test, $N = 4$ independent experiments (IGF1), 2 independent experiments (water); data shown are mean ± SEM).
E Analysis of GFP-EB3 comet velocity after treatment with PPP or IGF1. PPP significantly reduced the average velocity of GFP-EB3 comets compared to controls (DMSO, 91 EB3 comets, 10 axons; PPP, 138 comets, 23 axons; ***$P = 2.6 \times 10^{-10}$, $N = 4$ independent experiments, Student's t-test). IGF1 increased the growth rate of GFP-EB3 comets compared to controls (water, 85 comets, 14 axons; IGF1, 213 comets, 25 axons; ***$P = 1.7 \times 10^{-10}$, $N = 4$ independent experiments, Student's t-test; boxplot shows median, first and third quartiles. Upper/lower whiskers extend to 1.5 * the interquartile range).

© 2020 The Authors

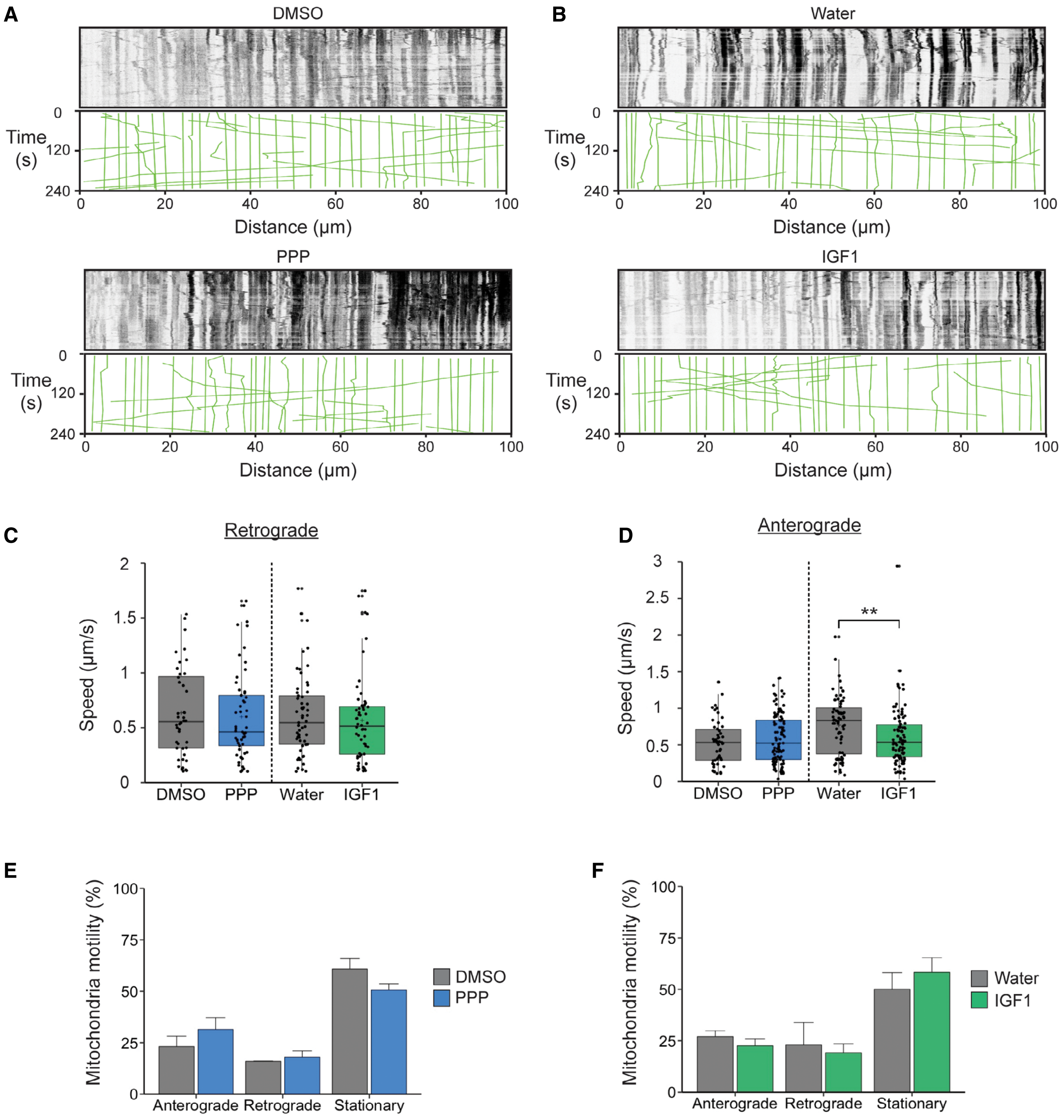

Figure EV5.

 © 2020 The Authors

Figure EV5. IGF1R activity does not impact on the axonal trafficking of mitochondria.

A Kymographs of TMRM-labelled mitochondria in DIV 6 PMN after treatment with 1 μM PPP or DMSO for 45 min.

B Kymographs of TMRM-labelled mitochondria in DIV 6 primary motor neurons after treatment with 50 ng/ml IGF1 or water for 45 min.

C IGF1R modulation does not impact on the retrograde transport of mitochondria (DMSO: 0.64 ± 0.06 μm/s, 45 organelles, N = 2 independent experiments; PPP: 0.60 ± 0.05 μm/s, 57 organelles, N = 3 independent experiments; P = 0.66, Student's t-test; water: 0.61 ± 0.05 μm/s, 57 organelles, N = 3 independent experiments; IGF1: 0.57 ± 0.05 μm/s, 56 organelles, N = 3 independent experiments, P = 0.56, Student's t-test; boxplot shows median, first and third quartiles. Upper/lower whiskers extend to 1.5 * the interquartile range).

D Effect of IGF1R treatment on anterograde mitochondrial velocity. PPP caused no significant change in transport rates (DMSO: 0.54 ± 0.04 μm/s, 50 organelles, N = 2 independent experiments; PPP: 0.59 ± 0.03 μm/s, 105 organelles, N = 3 independent experiments; P = 0.32, Student's t-test). In contrast, IGF1 treatment significantly slowed down anterograde mitochondria moving anterogradely (water: 0.76 ± 0.05 μm/s, 75 organelles, N = 3 independent experiments; IGF1: 0.60 ± 0.04 μm/s, 98 organelles; **P = 0.009, Student's t-test, N = 3 independent experiments; boxplot shows median, first and third quartiles. Upper/lower whiskers extend to 1.5 * the interquartile range).

E Quantification shows that the directionality of mitochondria transport after PPP treatment is not affected (anterograde: DMSO, 23.17 ± 5.0%, 93 organelles, N = 2 independent experiments; PPP, 31.46 ± 5.6%, 177 organelles, N = 3 independent experiments, P = 0.35; retrograde: DMSO, 16.02 ± 0.13%, 64 organelles N = 2 independent experiments, PPP, 17.98 ± 3.0%, 105 organelles, N = 3 independent experiments, P = 0.58; stationary: DMSO, 60.8 ± 5.1%, 248 organelles, N = 2 independent experiments; PPP, 50.55 ± 3.1%, 291 organelles, N = 3 independent experiments, P = 0.24, Student's t-test; data shown are mean ± SEM).

F Quantification shows that IGF1 treatment does not alter the directionality of mitochondria (anterograde: water, 26.99 ± 2.9%, 119 organelles, N = 3 independent experiments; IGF1, 22.58 ± 3.4%, 136 organelles, N = 3 independent experiments, P = 0.39; retrograde: water, 22.94 ± 10.9%, 95 organelles, N = 3 independent experiments; IGF1, 19.2 ± 4.3%, 115 organelles, P = 0.79; stationary: water, 50.1 ± 8.0%, 222 organelles, N = 3 independent experiments; IGF1, 58.25 ± 7.1%, 340 organelles, N = 3 independent experiments, P = 0.51, Student's t-test; data shown are mean ± SEM).

© 2020 The Authors
