# Supplementary figures and images for: IGF1R regulates retrograde axonal transport of signalling endosomes in motor neurons
Source: EMBO Rep. 2020 Feb 7;21(3):e49129. doi: 10.15252/embr.201949129 (PMC7054680; doi:10.15252/embr.201949129)

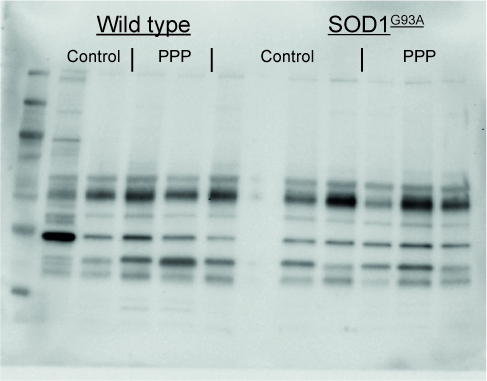

Supplement: Supplementary file 11 — Source Data for Appendix [file EMBR-21-e49129-s012.zip › embr201949129-sup-0012-SDataAppendixFigS7.tif]

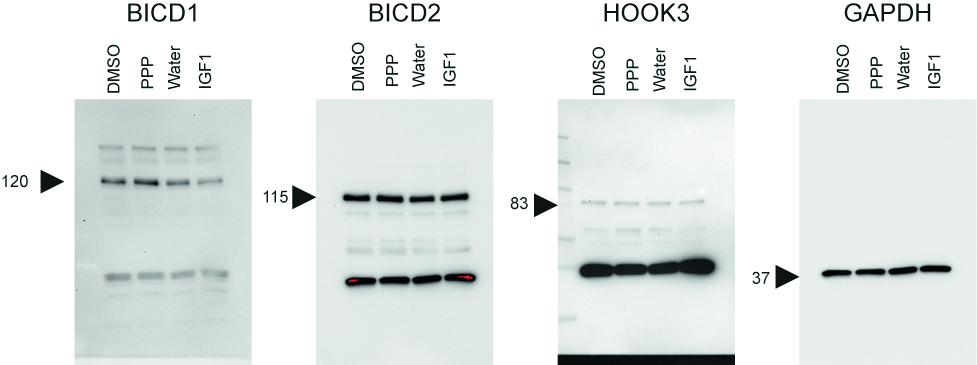

Supplement: Supplementary file 13 — Source Data for Figure 5 [file EMBR-21-e49129-s011.tif]
